# Small Molecules with Similar Structures Exhibit Agonist, Neutral Antagonist or Inverse Agonist Activity toward Angiotensin II Type 1 Receptor

**DOI:** 10.1371/journal.pone.0037974

**Published:** 2012-06-14

**Authors:** Shin-ichiro Miura, Yoshihiro Kiya, Hiroyuki Hanzawa, Naoki Nakao, Masahiro Fujino, Satoshi Imaizumi, Yoshino Matsuo, Hiroaki Yanagisawa, Hiroyuki Koike, Issei Komuro, Sadashiva S. Karnik, Keijiro Saku

**Affiliations:** 1 Department of Cardiology, Fukuoka University School of Medicine, Fukuoka, Japan; 2 Department of Molecular Cardiology, Lerner Research Institute, The Cleveland Clinic Foundation, Ohio, United States of America; 3 Research & Development Division, Daiichi Sankyo Company, Ltd., Tokyo, Japan; 4 Department of Cardiovascular Medicine, Osaka University Graduate School of Medicine, Suita, Japan; Medical School of Hannover, United States of America

## Abstract

Small differences in the chemical structures of ligands can be responsible for agonism, neutral antagonism or inverse agonism toward a G-protein-coupled receptor (GPCR). Although each ligand may stabilize the receptor conformation in a different way, little is known about the precise conformational differences. We synthesized the angiotensin II type 1 receptor blocker (ARB) olmesartan, R239470 and R794847, which induced inverse agonism, antagonism and agonism, respectively, and then investigated the ligand-specific changes in the receptor conformation with respect to stabilization around transmembrane (TM)3. The results of substituted cysteine accessibility mapping studies support the novel concept that ligand-induced changes in the conformation of TM3 play a role in stabilizing GPCR. Although the agonist-, neutral antagonist and inverse agonist-binding sites in the AT_1_ receptor are similar, each ligand induced specific conformational changes in TM3. In addition, all of the experimental data were obtained with functional receptors in a native membrane environment (in situ).

## Introduction

Over the past several decades, an important subject of research has been how molecules (agonist, neutral antagonist, and inverse agonist) bind to and change the conformation to activate or inactivate G-protein coupled receptors (GPCRs) [1], [2]. Although only light-sensitive rhodopsin has been crystallized in different conformational states [3]–[5], the crystal structures of the ß-adrenergic receptors (ARs) ß_ 1_ and ß_ 2_-AR have recently been obtained for complexes of the receptors bound to inverse agonists [6], [7]. More recently, Rosenbaum *et al*. [8], Rasmussen *et al.* [9], and Warne *et al*. [10] reported that structural, biophysical and computational studies could provide insight into the agonist-induced activation of ß_1_ and ß_2_-AR. Based on a comparison of the agonist-induced crystal structure [8] to that in which ß_2_-AR is bound to an inverse agonist [7], it has been shown that the transition between the R* (active) state and R (inactive) state involves the repacking of hydrophobic amino acid residues with slight rotations of transmembrane (TM) 5 and 6. Nonetheless, the conformation range and dynamics of the effects of ligands on GPCRs may differ from one receptor to another.

Angiotensin II (Ang II) type 1 (AT_1_) receptor, which is a member of the GPCR superfamily, has a widespread tissue distribution and mediates most known cardiovascular functions including vasoconstriction, cardiovascular hypertrophy and hyperplasia [11]. The AT_1_ receptor exhibits a low level of constitutive activity in the absence of any ligand [12]. The data indicate that a small portion of the receptor is in the active state (R*). The blockade of constitutive activity may confer resistance to diverse effects. Inverse agonists inhibit basal constitutive activity, and we previously reported that the AT_1_ receptor blocker (ARB) olmesartan showed inverse agonism toward inositol phosphate (IP) production [13]. We revealed that cooperative interactions between the hydroxyl group and Tyr^113^ (TM3) and between the carboxyl group and His^256^ (TM6) are crucial for the potent inverse agonist activity of olmesartan [13]. In addition, the olmesartan-related compound R239470, which has a non-acidic carbamoyl group instead of a carboxyl group, acts as a neutral antagonist. Each inverse agonist, neutral antagonist and agonist may induce a specific conformational change in TM3 as well as TM6 in the AT_1_ receptor. However, little is known about this topic, even though it should promote our understanding of the structural basis of ligand-receptor interaction.

Small differences in the chemical structures of ligands are responsible for inducing agonism, neutral antagonism or inverse agonism in a GPCR [1], [14], [15]. Although each agonist, neutral antagonist or inverse agonist may stabilize the receptor conformation in a different way, little is known about the precise differences in the AT_1_ receptor. Specifically, little is known about the structural basis for the functional versatility of the AT_1_ receptor, which switches from the R state to the R* state under the influence of ligands. Since the AT_1_ receptor has not yet been crystallized, we used an alternate approach in this study. Structure-based drug-design methods rely on knowledge about the molecules that bind to the biological target AT_1_ receptor and the focus is on ligands, and is usually referred to as receptor-based drug design [16]–[18]. In this approach, ligand molecules are built-up within the constraints of the binding pocket by assembling small pieces in a stepwise manner. In the present case, since amino-aromatic bonding between the agonist “switch” Tyr^4^ of Ang II and the agonist switch-binding residue Asn^111^ of the AT_1_ receptor is responsible for initiating receptor activation [19], we speculated that aromaticity of the ligand as an agonist may be important for the activation of AT_1_ receptor. The neutral antagonist R239470 with an aromatic ring may act as an agonist.

Using the substituted cysteine accessibility mapping (SCAM) method [20]–[22], Martin *et al*. identified the residues within TM3 of the AT_1_ receptor that contribute to the formation of the binding site pocket and found that constitutive activation of AT_1_ receptor causes slight counterclockwise rotation of TM3 [20]. We speculated that an inverse agonist, neutral antagonist and agonist would induce different changes in the conformation of TM3 of AT_1_ receptor. To address this question, we systematically mutated the AT_1_ receptor and examined whether olmesartan-related compounds would induce agonism, and subsequently analyzed the specific action of an inverse agonist, neutral antagonist and agonist on the receptor conformation with respect to stabilization around TM3 as assessed by SCAM and molecular modeling of the AT_1_ receptor.

Based on the results of these experiments, we developed a new agonist from the inverse agonist against the AT_1_ receptor and demonstrated the ligand-induced specific action on changes in the conformation of TM3 in the receptor.

## Results

### Synthesis of Olmesartan-related Compounds

**Figure 1:**
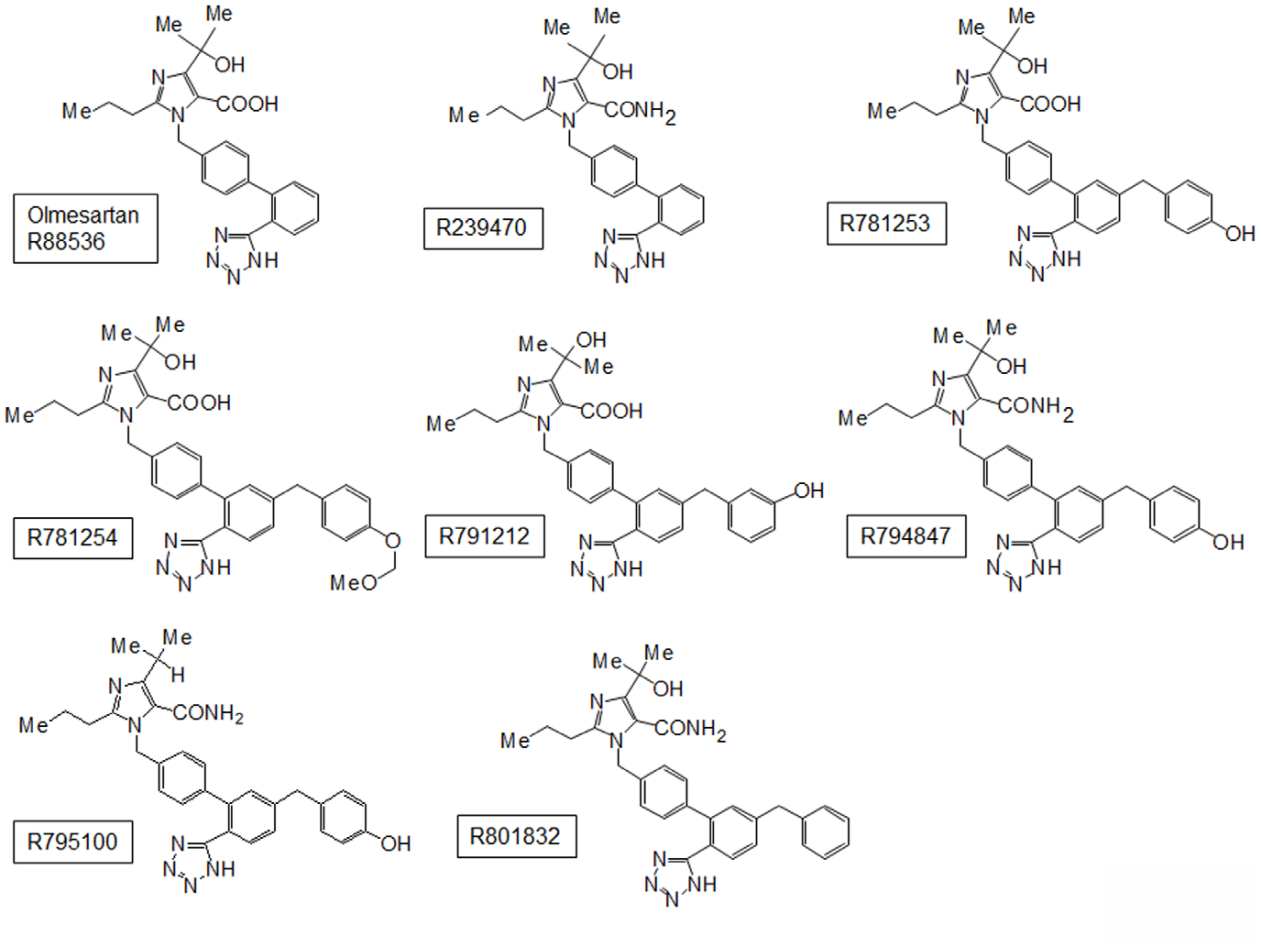
Construction of olmesartan-related compounds.

As shown in Fig. 1, we syntesized olmesartan-related compounds (R781253, R781254, R791212, R794847, R795100 and R801832) as well as R239470. To identify an agonist for AT_1_ receptor, we initially syntesized R781253, which retains critical side-chains of olmesartan (a carboxyl group and a hydroxyl group) and has an additional 4-hydroxybenzyl (-CH_2_C_6_H_4_OH) group in a biphenyltetrazole scaffold because phenol in Tyr^4^ (Ang II) - Asn^111^ (AT_1_ receptor) interaction is a trigger for receptor activation [19]. Next, we syntesized R781254, which contains a methoxymethyl ether (-OCH_2_OMe) to understand the importance of the hydroxyl group of phenol. R791212 has a 3-hydroxybenzyl group instead of the 4-hydroxybenzyl group in R781253. In addition, R794847 has a carbamoyl group instead of a carboxyl group in the imidazole ring of R781253. Next, we syntesized R795100, which contains an isopropyl group and a carbamoyl group instead of a hydroxyisopropyl group and carboxyl group, respectively. Finally, we syntesized R801832 which has a structure similar to R794847, except for lacking the phenolic hydroxyl group.

### Pharmacological Properties of Olmesartan and Related Compounds

First, we examined whether the various olmesartan-related compounds retained the ability to bind to AT_1_- wild-type (WT) and N111G mutant receptors (Table 1). Since we previously reported that the AT_1_-N111G mutant receptor had high basal activity in the absence of Ang II [23], [24] and could be used to determine the inverse agonism of olmesartan, we also analyzed the binding affinities of olmesartan-related compounds in the mutant receptor. The affinities of R791212, R794847 and R795100 in the AT_1_-WT receptor were 10-fold less than that of olmesartan. In addition, the affinities of R781254 and R801832 were reduced by 100-fold, which suggests that hydroxyphenyl moiety in the biphenyltetrazole scaffold in olmesartan-related compounds prevents binding to the AT_1_-WT receptor. On the other hand, all of the olmesartan-related compounds showed higher binding affinities, but <2-fold compared with olmesartan, in AT_1_-N111G receptor, suggesting that binding to the mutant receptor was insensitive to the modification of olmesartan because the conformation of the receptor is constitutively active.

**Table 1:** Binding affinities (*K*_d_) of Ang II and R compounds to AT_1_ wild-type (WT) and mutant receptors.

Ang II & ARBs	WT	N111G
	*nM*	
Ang II	1.2±0.5	0.6±0.2
Olmesartan	2.3±0.8 (1.0)	40±19 (1.0)
R239470	0.8±0.3 (0.3)	15±21 (0.4)
R781253	21±10 (9.1)	52±38 (1.3)
R781254	396±80 (172)	73±21 (1.8)
R791212	125±26 (54)	33±8 (0.8)
R794847	48±12 (21)	35±13 (0.9)
R795100	179±84 (78)	52±15 (1.3)
R801832	276±22 (120)	15±6 (0.4)

**Figure 2:**
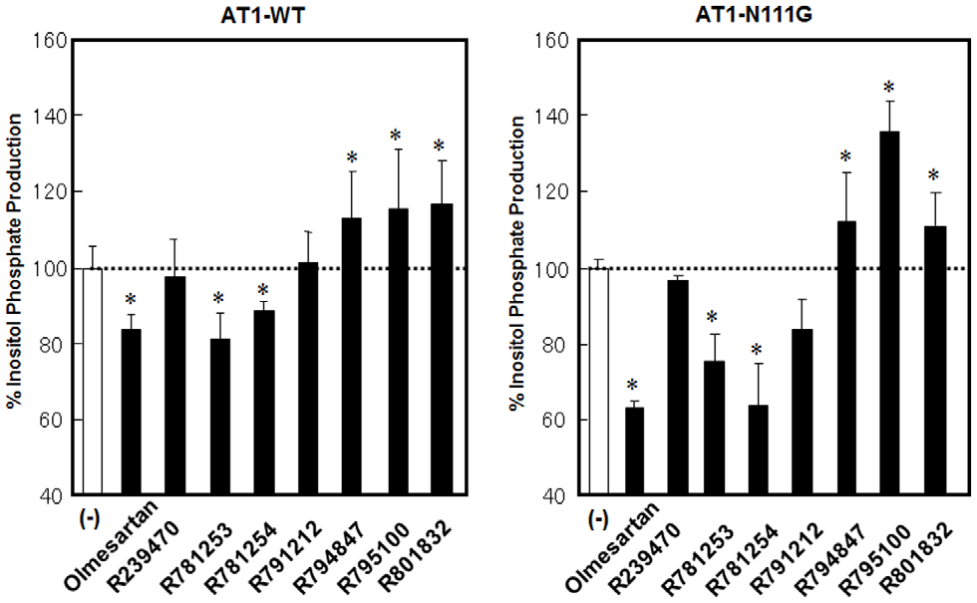
% inositol phosphate (IP) production with or without 1 µM of olmesartan and olmesartan-related compounds in COS1 cells transiently expressing the wild-type (WT) and N111G AT_1_ receptor. The test compounds were added 1 h before the measurement of IP. 100% IP production indicates basal IP production in WT (1,033±149 cpm) and N111G (2,087±414 cpm) AT_1_ receptor-transfected cells. Ang II (0.1 µM)-induced maximum IP production in WT and N111G AT_1_ receptor-transfected cells were 4,037±479 cpm and 4,138±362 cpm, respectively. n=4–7, *p<0.05 vs. no treatment.

Next, we analyzed whether the olmesartan-related compounds induced agonism toward inositol phosphate (IP) production on the AT_1_-WT and N111G receptors (Fig. 2). R781253 and R781254 retain inverse agonism. After modification of the phenolic group of R781253, R791212 lost inverse agonism and showed neutral antagonism. Interestingly, R794847 lost neutral antagonism, and showed agonism, suggesting that modification of the carboxyl group to the carbamoyl group in addition to insertion of a hydroxyphenyl group in the biphenyltetrazole scaffold induced agonism. Although R795100 and R801832 showed agonism, the strength of these agonism were similar to that of R794847. All of the olmesartan-related compounds showed similar degrees of neutral antagonism and agonism toward the AT_1_-N111G receptor compared with the pharmacological behaviors of these compounds in the AT_1_-WT receptor. We selected R794847 as an agonist. Thus, in subsequent analyses, olmesartan was used as an inverse agonist, R239470 was a neutral antagonist, and R794847 was an agonist.

### General Strategy Used to Identify AT_1_-WT Receptor Contact Sites

To detect specific contact sites between olmesartan, R239470 or R794847 and AT_1_-WT receptor in TM3, we mutated 13 amino acid residues from Ser^105^ to Phe^117^ (S105A, A106G, S107A, V108A, S109A, F110A, L112A, Y113F, Y113A, A114G, S115A, V116A and F117A) and examined the binding affinities of these compounds toward AT_1_-WT and mutant receptors (Table 2). The expression levels of the AT_1_-WT and mutant receptors (point mutations between Ser^105^ and Phe^117^) were within the same order of magnitude (Table S1). The affinities of Ang II were almost the same in all mutants, within <2-fold, compared to AT_1_-WT receptor. The affinity of olmesartan was reduced by >10-fold in L112A and Y113F mutants compared with the AT_1_-WT receptor, suggesting that Leu^112^ and Tyr^113^ in the AT_1_ receptor are involved in binding to olmesartan. R239470, which has a chemical structure similar to that of olmesartan but has a carbamoyl group instead of a carboxyl group, exhibited a >10-fold reduction in binding affinity to the L112A (54-fold reduction), Y113F (21-fold reduction) and Y113A (183-fold reduction) mutants compared with the AT_1_-WT receptor. R794847, which has a carbamoyl group instead of a carboxyl group in olmesartan with a phenyl group, also exhibited a 8.1-fold reduction in affinity in the Y113F mutant, and a >10-fold reduction in affinity in the A106G (17-fold reduction), V108A (51-fold reduction), Y113A (38-fold reduction) and V116A (45-fold reduction) mutants. These results suggest that Leu^112^ and Tyr^113^ are essential for binding between R239470 and the AT_1_-WT receptor. Moreover, Ala^106^, Val^108^, Leu^112^, Tyr^113^ and Val^116^ may be critical sites for the binding of R794847 to the AT_1_-WT receptor. While both R239470 and R794847 have a carbamoyl group, only R794847, which showed agonism, has a hydroxyphenyl group, indicating that Ala^106^ and Val^116^ in the AT_1_ receptor are probably important for binding to R794847 and induce agonism.

**Table 2:** Binding affinities (*K*_d_) of Ang II, olmesartan, R239470 and R794847 to AT_1_ wild-type (WT) and mutant receptors.

Receptor	*K_d_*			
	Ang II	Olmesartan	R239470	R794847
	*nM*			
WT	1.2±0.5 (1.0)	2.3±0.8 (1.0)	0.8±0.3 (1.0)	48±12 (1.0)
S105	0.6±0.2 (0.5)	6.7±3.8 (1.8)	3.2±2.0 (4.0)	37±23 (0.8)
A106G	0.9±0.2 (0.8)	6.1±2.2 (2.6)	4.8±1.5 (6.0)	792±202 (17)
S107A	0.5±0.1 (0.4)	2.5±0.5 (1.1)	1.3±0.2 (1.6)	59±27 (1.2)
V108A	0.6±0.2 (0.5)	4.8±2.0 (2.1)	1.6±0.8 (2.0)	2448±1314 (51)
S109A	0.9±0.6 (0.8)	2.5±0.8 (1.1)	0.9±0.2 (1.1)	34±11 (0.7)
F110A	0.8±0.3 (0.7)	2.6±0.5 (1.1)	1.2±0.3 (1.5)	68±19 (1.4)
L112A	0.9±0.3 (0.8)	27±7 (12)	43±12 (54)	31±5 (0.6)
Y113F	1.8±1.0 (2.6)	52±7 (23)	17±3 (21)	388±54 (8.1)
Y113A	0.7±0.2 (1.0)	12±3 (5.2)	146±15 (183)	1804±205 (38)
A114G	0.8±0.1 (0.7)	4.0±1.4 (1.7)	1.1±0.1 (1.4)	183±17 (3.8)
S115A	1.2±0.1 (1.0)	3.1±1.7 (1.3)	1.3±0.4 (1.6)	150±41 (3.1)
V116A	1.1±0.4 (0.9)	8.8±1.2 (3.8)	2.6±0.7 (3.2)	2154±802 (45)
F117A	0.8±0.1 (0.7)	2.5±1.4 (1.1)	1.5±0.04 (1.9)	54±19 (1.1)

### Generation of Cysteine-substituted Mutant AT_1_ Receptor

To clarify the olmesartan-related compound-induced helical movement of TM3 on the AT_1_ receptor, we introduced cysteine substitutions into the region of TM3 that is known to be critical for receptor activation [19] before performing a series of SCAM experiments (Fig. S1).

As we reported previously [21], the percentage (%) inhibition of ^125^I-[Sar^1^, Ile^8^] AngII binding by methanethiosulphonate ethyl-ammonium (MTSEA^+^) reagent was approximately 30% on AT_1_ receptor, since Cys^76^ in TM2 was accessible to water within the ligand pocket. In addition, mechanical stretching increased the accessibility of Cys^289^ by inducing a change in the conformation of TM7 [22]. To differentiate between single and multiple movements of one or more native cysteines, we created TM3 residues that were substituted with cysteine one at a time from Ser^105^ to Phe^117^ using the C76A/C289A mutant genetic background and evaluated each triple mutant for MTSEA sensitivity.

We examined whether the various cysteine-substituted mutants retained the ability to bind AT_1_ receptor using radioligand binding studies (Table 3). The expression levels of the AT_1_-WT and mutant receptors substituted with cysteine were within the same order of magnitude (Table S1). Binding studies with membranes prepared from receptor-expressing cells showed that all AT_1_ mutant receptors were able to bind Ang II with high affinity. Olmesartan, R239470 and R794847 showed higher binding affinities toward all of the AT_1_ mutant receptors except for C76A/Y113C/C289A mutant, which was not used in further analysis.

**Table 3:** Binding affinities (*K*_d_) of Ang II, olmesartan, R239470 and R794847 to AT_1_ wild-type (WT) and mutant receptors.

Receptor	*K_d_*			
	Ang II	Olmesartan	R239470	R794847
	*nM*			
WT	1.2±0.5 (1.0)	2.3±0.8 (1.0)	0.8±0.3 (1.0)	48±12 (1.0)
C76A/C289A	1.3±0.3 (1.1)	5.7±1.6 (2.5)	2.4±0.5 (3.0)	73±20 (1.5)
C76A/A106C/C289A	1.2±0.3 (1.0)	8.9±4.0 (3.9)	4.1±1.0 (5.1)	193±73 (4.0)
C76A/S107C/C289A	1.2±0.3 (1.0)	6.2±3.2 (2.7)	1.8±0.4 (2.3)	102±19 (2.1)
C76A/V108C/C289A	1.5±0.6 (1.3)	32±1 (14)	10±2 (13)	391±54 (8)
C76A/S109C/C289A	1.3±0.3 (1.1)	29±5 (13)	11±3 (14)	96±18 (2.0)
C76A/F110C/C289A	0.6±0.1 (0.5)	6.6±2.4 (2.9)	2.4±1.1 (3.0)	126±13 (2.6)
C76A/N111C/C289A	1.4±0.2 (1.2)	12±4 (5.2)	5.4±3.0 (6.8)	180±94 (3.8)
C76A/L112C/C289A	1.6±0.3 (1.3)	6.1±2.1 (2.7)	3.2±1.3 (4.0)	62±9 (1.3)
C76A/Y113C/C289A	7.8±2.8 (6.5)	570±197 (248)	272±129 (340)	6419±2575 (134)
C76A/A114C/C289A	1.3±0.2 (1.0)	5.5±2.6 (2.4)	1.6±0.7 (2.0)	128±15 (2.7)
C76A/S115C/C289A	1.7±0.4 (1.4)	6.5±1.8 (2.8)	4.2±1.6 (5.3)	657±114 (14)
C76A/V116C/C289A	0.8±0.4 (0.7)	6.2±1.7 (2.7)	2.5±1.0 (3.1)	39±3 (0.8)
C76A/F117C/C289A	0.6±0.4 (0.5)	2.4±1.3 (1.0)	2.2±0.2 (2.8)	29±9 (0.6)

### Determination of Changes in the Conformation of TM3 in the AT_1_ Receptor

**Figure 3:**
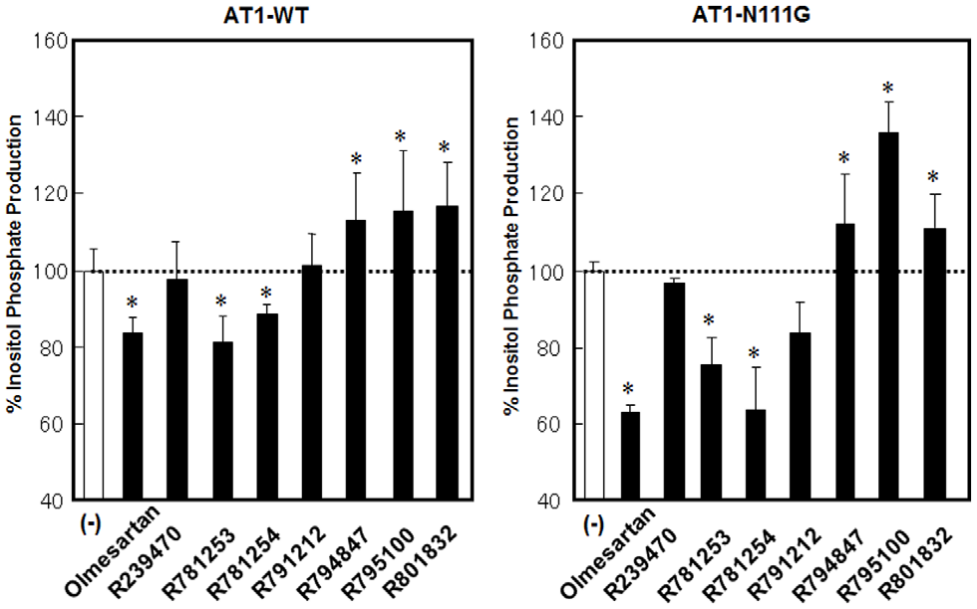
Changes in cysteine accessibility in COS1 cells expressing C76A/C289A mutant receptors in which the transmenbrane 3 residues from Ala^106^ to Phe^117^, except for position 113, were successively replaced by cysteine. 1 µM of ARBs, n=4–9, *p<0.05 vs. no treatment. #p<0.05 vs. C76A/C289A.

We found that olmesartan, R239470 and R794847 acted as an inverse agonist, a neutral antagonist and an agonist, respectively. Therefore, we investigated the agonist-, neutral antagonist- and inverse agonist-specific changes in receptor conformation with regard to stabilization around TM3. Differences in cysteine accessibility by ligand (-), olmesartan, R239470 and R794847 in AT_1_-WT and mutant receptors are shown in Fig. 3. The % inhibition of ^125^I-[Sar^1^, Ile^8^] AngII binding with the unliganded/unoccupied receptor [ligands (-)] in the A106C, V108C, S109C, N111C, L112C, S115C, V116C and F117C mutants in the C76A/C289A background were significantly higher than those in the C76A/C289A mutant with ligand (-), indicating that Ala^106^, Val^108^, Ser^109^, Asn^111^, Leu^112^, Ser^115^, Val^116^ and Phe^117^ face toward the ligand pocket of Ang II. In particular, the % inhibitions of the C76A/V108C/C289A and C76A/L112C/C289A mutants with ligand (-) were very high. Val^108^ and Leu^112^ definitely entered the ligand pocket.

There were no significant differences in the % inhibition of ^125^I-[Sar^1^, Ile^8^] Ang II binding in the A106C, V108C, N111C and S115C mutants in the C76A/C289A background among olmesartan, R239470 and R794847. Different values of % inhibition were seen between the ligands in the S109C, L112C, V116C and F117C mutants in the C76A/C289A background. For example, Leu^112^ shifts to further out of the ligand pocket with olmesartan and R239470 than with ligand (-), since the % inhibition in the C76A/L112C/C289A mutant with olmesartan or R239470 was significantly lower than that with ligand (-). Moreover, Leu^112^ shifts to further out of the ligand pocket with R794847 than with olmesartan or R239470, since the % inhibition in the C76A/L112C/C289A with R794847 was significantly lower than those with olmesartan and R239470.

**Figure 4:**
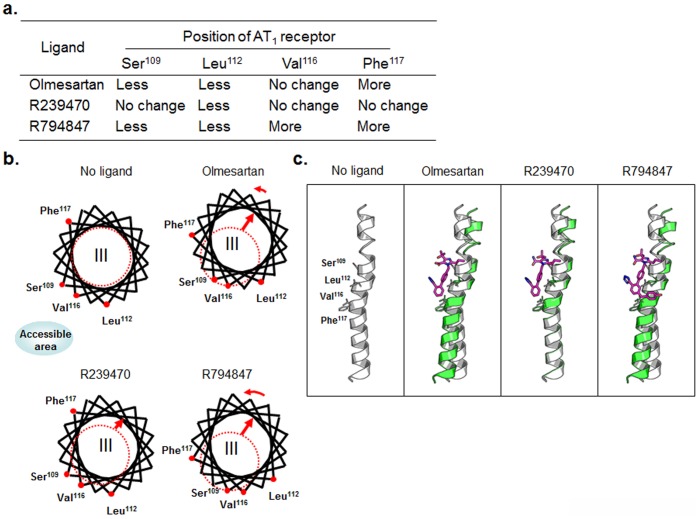
A. Summary of cysteine accessibilityat Ser^109^, Leu^112^, Val^116^ and Phe^117^ with olmesartan, R239470 and R794847 compared to ligand (-). Less and More indicate that the AT_1_ receptor is less or more accessible to the ligand pocket, respectively. B. Helical wheel representation of transmenbrane (TM)3 cysteine residues for ligand (-), olmesartan, R239470 and R794847. Olmesartan induced the outward displacement and counterclockwise rotation of TM3. Dotted circle indicates the original position of TM3 without ligand. Accessible area indicates a site of ligand pocket. C. Schematic view of TM3 movement derived from the SCAM results. TM3 without ligand is shown as a gray ribbon and TM3 with various ligands is represented as green ribbons. The ligands are colored magenta. (a) without ligand, (b) olmesartan, (c) R239470, (d) R794847.

We summarized whether Ser^109^, Leu^112^, Val^116^ and Phe^117^ become more or less accessible in the ligand pocket with olmesartan, R794847 or R239470 compared to ligand (-) (Fig. 4a). Since olmesartan showed less accessibility at Ser^109^ and Leu^112^, TM3 was shifted outward from the ligand pocket (Fig. 4bc). In addition, since olmesartan induced greater accessibility at Phe^117^, TM3 might show slight counterclockwise rotation. R239470 only induced a small conformational change in TM3. Since only Leu^112^ was less accessible with R239470, TM3 may be only slightly shifted outward from the ligand pocket. R794847 showed conformational changes similar to those with olmesartan. Since R794847 showed less accessibility at Ser^109^ and Leu^112^ and more accessibility at Val^116^ and Phe^117^, TM3 was shifted outward from the ligand pocket and showed greater counterclockwise rotation than with olmesartan.

### Molecular Modeling of the Interaction between ARBs and the AT_1_ Receptor

**Figure 5:**
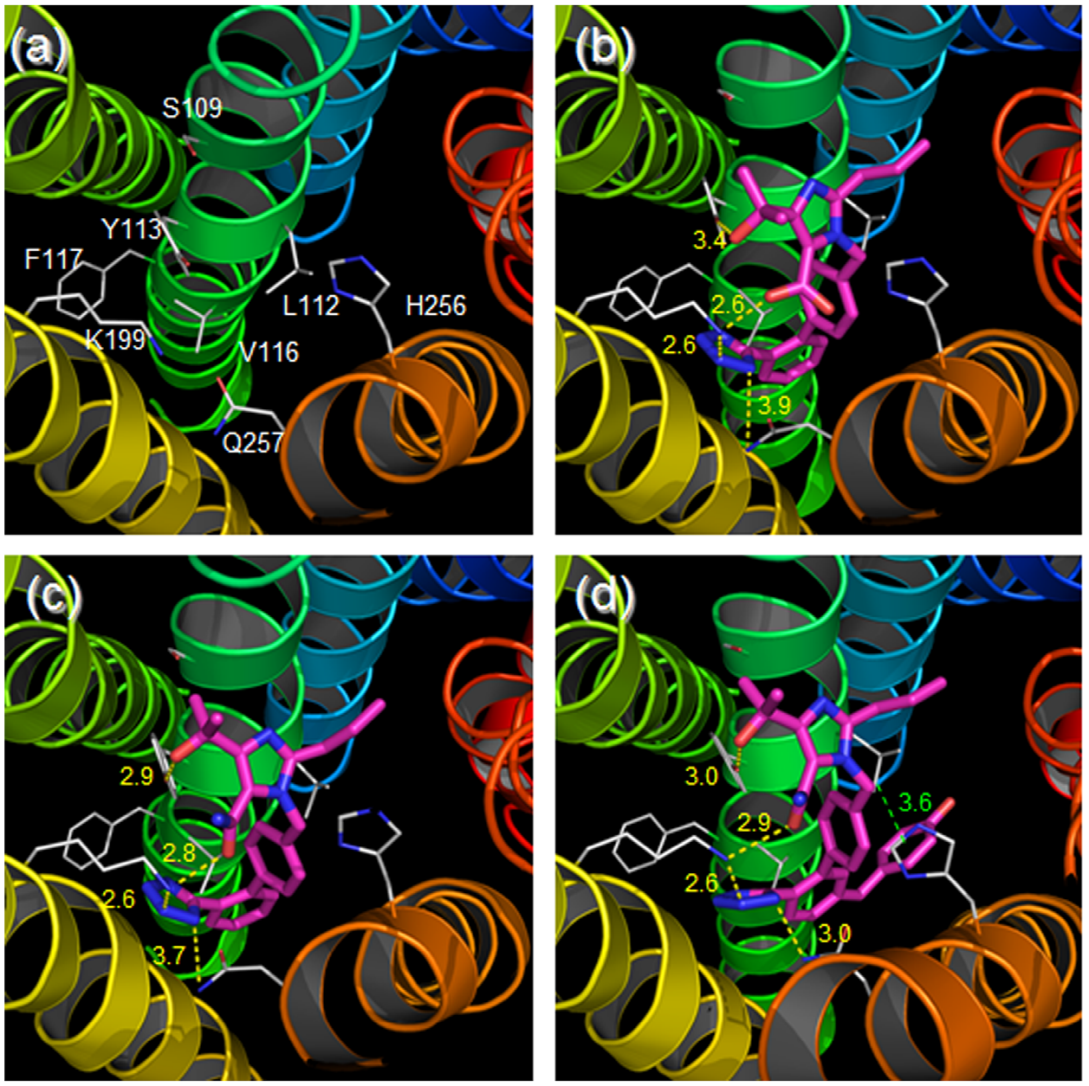
Putative binding mode of various ligands in AT_1_ receptor. Transmenbrane (TM)1–7 are shown as colored ribbons: blue (TM1), cyan (TM2), green (TM3), lime green (TM4), yellow (TM5), orange (TM6) and red (TM7). Sidechains of the focused residues are shown as gray lines, and ligands are represented as magenta sticks. The hydrogen bonds and electrostatic interactions are indicated by yellow dotted lines. The distance between Leu^112^ and the hydroxyphenyl moiety of R794847 is represented as a green dotted line. The unit of distance was Å. (a) without ligand, (b) olmesartan, (c) R239470, (d) R794847.

A molecular model was constructed based on a consideration of the interactions between the AT_1_ receptor and olmesartan, R239470 or R794847 as suggested from the mutation experiments and the changes in the conformation of TM3 in the AT_1_ receptor induced by olmesartan, R239470 and R794847 using the SCAM study (Fig. 5). The rotation and tilt of the TM helices are introduced as described in the Methods. The distances between Tyr^113^ in the AT_1_ receptor and the hydroxyl group of olmesartan and between Lys^199^ and the carboxyl group are 3.4 and 2.6 Å, respectively, which are reasonable distances for contributing to electrostatic and/or hydrogen bond interactions. R239470 showed slight changes in these distances (2.9 and 2.8 Å, respectively). In addition, the distances with R794847 were similar to those with R239470 (3.0 and 2.9 Å, respectively). Although R794847 did not show the reduction of binding affinity toward L112A mutant (31±5 nM) compared with WT (48±12 nM), Leu^112^ was closer to the centroid of the phenol ring of R794847 (3.6 Å) according to molecular modeling. In addition, the other compounds do not have a phenol ring in this position.

## Discussion

In the present study, small molecules with similar structures, olmesartan, R239470 and R794847, acted as an inverse agonist, neutral antagonist and agonist, respectively. These molecules induced specific actions in TM3 of AT_1_ receptor. Notably, activation of most GPCRs induces a certain change in the conformation of TM3. Our site-directed mutagenesis and SCAM studies support the novel concept that ligand-induced changes in the conformation of TM3 play a role in GPCR activation in a native membrane environment (in situ).

Although it is now generally accepted that individual ligands can induce different receptor conformations [25], little is known about the actual differences at the molecular level. To gain information about the transition from an inactive receptor conformation to an active conformation, several different techniques have been developed and used over the past decade. While the crystal structures of GPCRs [4]–[10] obtained from the rhodopsin, opsin, and ß_1_ and ß_2_-AR systems have recently been described, the crystal structures of AT_1_ receptor have not been elucidated. In addition, despite the importance of these structures for GPCR research, crystallography has major limitations with regard to characterizing and understanding the physiological importance of receptors. Using a native membrane environment (in situ), we systematically mutated the AT_1_ receptor and examined the specific effects of olmesartan, R239470 and R794847 on the conformation of the AT_1_ receptor with respect to stabilization of TM3 as assessed by SCAM and molecular modeling of the AT_1_ receptor.

While activating ligands (agonists) stabilize receptor conformations that increase signaling through G proteins, inhibiting ligands (inverse agonists) stabilize other conformations that decrease the basal, agonist-independent level of signaling. Although olmesartan had inverse agonism, R239470, which has a non-acidic carbamoyl group instead of a carboxyl group of olmesartan, lost inverse agonism and acts as a neutral antagonist. The hydroxyphenyl moiety represents the difference between the chemical structures of R239470 and R794847, and R794847 but not R239470 showed agonism. Thus, it may be possible to predict where to modify a ligand to get inverse agonism or antagonism and where to modify a ligand to get agonism.

Interestingly, R794847 and olmesartan produced the same direction of rotation in TM3. The difference between R794847 and olmesartan was that there is more counterclockwise rotation with R794847 than with olmesartan. Most of the olmesartan contact sites in TM3 on the AT_1_ receptor that were identified in the inverse agonism state of the receptor were also observed following activation of the receptor by the agonist R794847. With regard to ß_2_-AR, many of the interactions between the agonist BI-167107 and the ß_2_-AR were similar to those observed with the inverse agonist carazolol [9]. The hydroxyphenyl moiety, which represents the difference between the chemical structures of olmesartan and R794847, interacts with Leu^112^ of the AT_1_ receptor. An agonist only has to disrupt one key intramolecular interaction that is needed to stabilize the inactive state, since constitutive receptor activity can result from single mutations of amino acids from different regions of GPCRs [26]. Asn^111^ of the AT_1_ receptor is responsible for initiating receptor activation by Ang II [19]. When R794847 interacts with Leu^112^, R794847 may change the position of Asn^111^. Disruption of these stabilizing interactions by R794847 changes the energy barrier for inducing active states. In addition, R794847 exhibited a 45-fold reduction in affinity in the V116A mutant. Since R794847 induced more counterclockwise rotation than olmesartan, Val^116^ was closer to hydroxyphenyl group of R794847 (Fig. S2). The hydroxyphenyl group and side-chain of Val^116^ may form a weak hydrophobic environment, and it may also need to stabilize the AT_1_ receptor. Thus, R794847 stabilized receptor conformations in the active state. While olmesartan and R794847 only showed slight differences in binding sites, the specific stabilization of TM3 induced by each ligand was sufficiently different to signify important differences in agonism.

When the agonist- and inverse agonist-bound structures in ß_2_-AR are compared [9], the largest change is observed in TM6, with 11.4Å movement of the helix at Glu^268^. We only analyzed TM3 and did not examine other TMs, including TM6. In addition, the largest differences in the ß_2_-AR relative to the inactive structure were found at the cytoplasmic face of the receptor, with outward displacement of TM5 and TM6 and inward movement of TM7 and TM3 [9]. In this study, we performed molecular modeling of the interaction between R794847 and the AT_1_ receptor based on SCAM results. Although we did not focus on the cytoplasmic face, TM3 with R794847 was rotated counterclockwise. Although the agonist- and inverse agonist-binding sites in the receptor are similar, each ligand may induce differential changes in cytoplasmic face of the receptor. In addition, Martin *et al*. [20] reported that constitutive activation of AT_1_ N111G receptor causes slight counterclockwise rotation of TM3. Agonist-activated AT_1_ receptor and the constitutively active mutant of the AT1 receptor showed the same counterclockwise rotation of TM3. However, since agonist and inverse agonist induced the same direction of rotation in TM3 in this study, the direction of rotation may not be important for the receptor activation. Although the mechanism by which agonists induce or stabilize these conformational changes likely differs for different ligands and for different GPCRs, a better understanding of the conformational changes in TM3 of AT_1_ receptor might provide new avenues for drug design. In addition, since GPCR-targeted therapies include agonists as well as antagonists, these structures should have a broader impact in biological chemistry and pharmacology.

The movements of TMs 3 and 6 at the cytoplasmic side of the membrane play an important role in the activation of G-protein-coupled receptors [27], [28]. For exsample, the salt bridge between the highly conserved Arg^139^ in TM3 and Glu^285^ in TM6 (the ionic lock) provides receptor stabilization [28]. R794847 might break the ionic lock and induce agonism in the AT_1_ receptor.

In summary, to the best of our knowledge, this is the first systematic and comprehensive analysis of receptor sites for three small molecules with similar structures. Furthermore, all of the experimental data were obtained with functional receptors present in a native membrane environment (in situ). Since GPCRs share a high degree of structural homology, our findings should be of broad general relevance.

## Materials and Methods

### Materials

The following antibodies and reagents were purchased or provided: the highly reactive, sulfhydryl-specific alkylating reagent CH_3_SO_2_-SCH_2_CH_2_NH_3_^+^ (methanethiosulfonyl ethyl-ammonium [MTSEA^+^], adduct size about 4.726 Å) (Toronto Research Chemicals Inc., Ontario, Canada); olmesartan-related compounds (Daiichi Sankyo Co., Tokyo, Japan); Ang II (Sigma-Aldrich, MO, USA); and ^125^I-[Sar^1^, Ile^8^] Ang II (Amersham Biosciences, Buckinghamshire, UK). The molecular structures of the ARBs which we synthesized are shown in Fig. 1.

### Mutagenesis and Expression of the AT_1_ Receptor and Membrane Preparation

The synthetic rat wild-type (WT) AT_1_ receptor gene, cloned in the shuttle expression vector pMT-3, was used for expression and mutagenesis studies (Table 1), as described previously [19].

### Cell Cultures, Transfections, Membrane Preparation

COS1 cells (African green monkey kidney fibroblast-like cell line, #CRL-1650, American Type Culture Collection, VA, USA) were cultured. The cells were maintained in 10% fetal bovine serum (FBS) and penicillin- and streptomycin-supplemented Dulbecco’s modified Eagle’s essential medium (Invitrogen) in 5% CO_2_ at 37°C. In the experiments, cells without cell-growth supplement were used. Cell viability in control experiments was >95% by trypan blue exclusion analysis. The WT and mutant AT_1_ receptors were transiently transfected into COS1 cells using Lipofectamine 2000 liposomal reagent (Roche Applied Science) according to the manufacturer’s instructions. Cell membranes were prepared by the nitrogen Parr bomb disruption method using 0.25 M sucrose solution in the presence of protease inhibitors.

### Competition Binding Study

The *K*d and *B*max values of receptor binding were determined by ^125^I-[Sar^1^, Ile^8^] AngII-binding experiments under equilibrium conditions, as described previously [19]. Membranes expressing the WT or mutant AT_1_ receptors were incubated with 100 pM ^125^I-[Sar^1^, Ile^8^] AngII for 1 h at 22°C in a volume of 125 µl. The binding reaction was terminated by filtering the incubation mixture through Whatman GF/C glass filters, and the residues were extensively washed further with binding buffer. The bound ligand fraction was determined from the counts per minute (cpm) remaining on the membrane. Binding kinetics values were determined as previously described [19].

### Substituted Cysteine Accessibility Mapping (SCAM) Study

The SCAM method has been used to probe relative changes in the conformation of constitutively active AT_1_ receptor [20]–[22] and other GPCRs [29], [30] by validating the presence of Cys residues within the ligand pocket. We used a highly reactive, sulfhydryl-specific reaction with methanethiosulfonyl ethyl-ammonium (MTSEA^+^), which reacts a billion times faster with water-exposed and ionized Cys than with lipid-exposed and un-ionized Cys. In this reaction, a positively charged sulfonylmethylammonium group is added to a water-exposed Cys. Aliquots of cell membranes are incubated with or without ARBs for 20 min in phosphate buffer at room temperature. The membranes are then diluted with same buffer and centrifuged. After washout the cell membranes, the membranes are incubated with 2.5 mM MTSEA^+^ at room temperature for 2 min in HEPES buffer (pH7.4). The Membranes are then diluted with same buffer to stop the reaction and centrifuged. One hundred µl aliquots were used to assay for ^125^I-[Sar^1^,Ile^8^]Ang II binding. The fractional inhibition is calculates 1 − [(specific binding after MTX reagent) / (specific binding without reagent)].

### IP Production Study

Cells expressing the WT and mutant AT_1_ receptors were incubated for 30 minutes at 22°C with or without the indicated concentrations of ARBs. The cells were washed-out once with the use of excess cold HBSS, and used in an IP production study. Total soluble IP was measured by the perchloric acid extraction method, as described previously [16].

### Molecular Modeling of AT_1_ Receptor-ARBs

For molecular modeling of the AT_1_ receptor with olmesartan, the initial structure was prepared as previously reported [11] followed by energy minimization. In addition, for a model of the AT_1_ receptor without a ligand, the ligand was removed from the final binding model of olmesartan as shown above, and then TM3 was manually tilted by 15 degrees, followed by energy minimization. In all the other binding models, the final model with olmesartan was used as an initial structure. For R239470, extracellular-half (Gly^97^-Tyr^113^) and intracellular-half (Ala^114^-Val^131^) of TM3 are tilted by 5 and 15 degrees, respectively, and rotated by 5 degrees in a clockwise direction. The ligand was then replaced by R239470, followed by energy minimization. For R794847, TM2 and TM3 were rotated by 10 degrees in a counterclockwise direction and TM6 and TM7 were moved away from the pocket manually by 1.4 Å for R794847. The ligand was then replaced by R794847, followed by energy minimization. Finally, the minimization was performed by MacroModel 9.7 or 9.8 (Schrödinger Inc). The force field was set to OPLS2005 with a GB/SA water solvation model and all of the atoms of the protein backbone were fixed during energy minimization. The final binding model was obtained by the Polak-Ribier Conjugate Gradient (PRCG) method with a convergence threshold of 0.005 for less than 5000 iterations.

### Statistical Analysis

The results are expressed as the mean ± standard deviation of three or more independent determinations. Significant differences in measured values were evaluated with an analysis of variance using Fisher’s t-test and a paired or unpaired Student’s *t*-test, as appropriate. Statistical significance was set at <0.05.

## Supporting Information

Figure S1 **Revised secondary-structure model of rat wild-type AT_1_ receptor based on the structure of bovine rhodopsin.** Extracellular Cys^18^-Cys^274^ and Cys^101^-Cys^180^ form disulfide bonds. The epitope tag attached to the C-terminal end that was used for detection by the ID4 monoclonal antibody is underlined. Bold residues indicate mutated sites.
(PPT)

Figure S2 **Putative binding mode of R794847 and Val^116^ of AT_1_ receptor.** Transmenbrans (TMs) are shown as colored ribbons: green (TM3), lime green (TM4), yellow (TM5) and orange (TM6). Yellow dots indicate a hydrophobic environment.
(PPT)

Table S1 **Maximal binding capacities (** ***B*** **_max_) of Ang II to AT_1_ wild-type (WT) and mutants receptors.**
(DOC)
