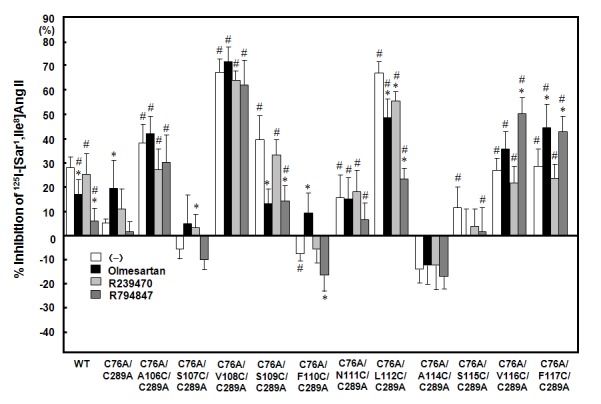# Correction: Small Molecules with Similar Structures Exhibit Agonist, Neutral Antagonist or Inverse Agonist Activity toward Angiotensin II Type 1 Receptor

**DOI:** 10.1371/annotation/158ee8e2-b9da-4d86-9085-457700f96b92

**Published:** 2013-05-03

**Authors:** Shin-ichiro Miura, Yoshihiro Kiya, Hiroyuki Hanzawa, Naoki Nakao, Masahiro Fujino, Satoshi Imaizumi, Yoshino Matsuo, Hiroaki Yanagisawa, Hiroyuki Koike, Issei Komuro, Sadashiva S. Karnik, Keijiro Saku

The version of Figure 3 included in the article is a duplicate of Figure 2. Please see the correct Figure 3 file here: 

**Figure:**