# Supplementary material for: Small Molecules with Similar Structures Exhibit Agonist, Neutral Antagonist or Inverse Agonist Activity toward Angiotensin II Type 1 Receptor
Source: PLoS One. 2012 Jun 14;7(6):e37974. doi: 10.1371/journal.pone.0037974 (PMC3375280; doi:10.1371/journal.pone.0037974)
Supplement: Figure S1 — Revised secondary-structure model of rat wild-type AT1 receptor based on the structure of bovine rhodopsin. Extracellular Cys18-Cys274 and Cys101-Cys180 form disulfide bonds. The epitope tag attached to the C-terminal end that was used for detection by the ID4 monoclonal antibody is underlined. Bold residues indicate mutated sites. (PPT) [file pone.0037974.s001.ppt]

## Slide 1
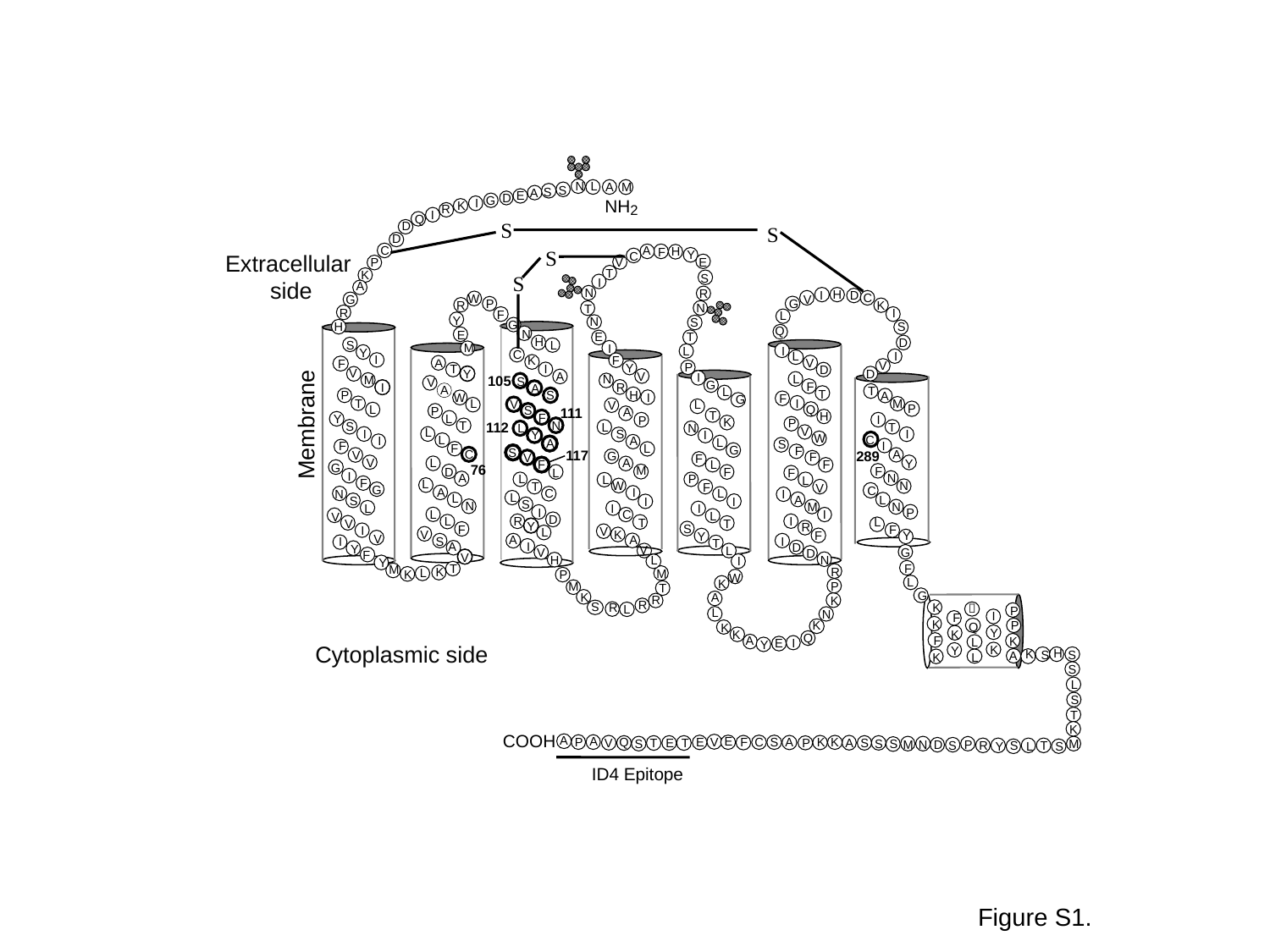

N
L
M
A
S
S
A
E
D
G
NH
 I
K
2
R
 I
S
Q
S
D
D
S
C
A
H
F
Y
C
Extracellular
 side
E
V
P
S
T
K
S
 I
A
N
R
H
 I
D
C
W
V
G
G
P
K
R
N
T
R
 I
F
L
Y
N
S
G
S
H
Q
N
E
E
T
H
D
S
L
M
 I
L
 I
Y
C
 I
L
 I
K
F
A
V
F
V
P
Y
T
I
D
V
105
Y
D
A
V
I
N
L
M
S
V
G
F
R
I
A
A
T
L
T
H
S
P
A
I
W
F
G
T
M
L
I
V
L
V
111
P
L
Q
S
P
A
T
H
F
Y
Membrane
L
I
112
P
K
P
N
T
S
L
T
L
N
V
L
 I
S
I
Y
I
W
L
C
A
 I
L
A
S
I
F
117
L
289
F
G
F
S
A
C
V
G
F
V
F
76
V
L
A
Y
F
L
F
G
M
F
D
F
L
F
 I
A
N
L
P
L
L
F
L
W
N
T
F
V
G
C
A
I
C
L
N
I
L
L
A
L
S
I
I
S
N
M
N
L
I
I
I
P
L
I
C
L
V
D
I
L
R
L
T
V
T
Y
R
S
F
F
I
V
L
V
K
F
Y
Y
V
A
A
I
S
I
T
 I
A
D
Y
V
L
G
V
D
F
V
N
H
L
I
Y
F
T
M
K
R
L
M
K
P
W
L
K
M
P
T
G
K
A
R
K
R
K
S
R
Ｉ
L
P
L
N
 I
F
K
P
K
Q
K
Y
K
K
Q
F
A
K
L
E
I
Y
Cytoplasmic side
K
Y
H
K
S
S
A
K
L
S
L
S
T
K
COOH
A
A
E
P
E
C
A
Q
V
F
S
K
K
P
S
A
V
T
E
T
M
S
S
S
M
N
D
P
S
R
T
S
Y
L
S
ID4 Epitope
Figure S1.
