# Supplementary figures and images for: Small Molecules with Similar Structures Exhibit Agonist, Neutral Antagonist or Inverse Agonist Activity toward Angiotensin II Type 1 Receptor
Source: PLoS One. 2012 Jun 14;7(6):e37974. doi: 10.1371/journal.pone.0037974 (PMC3375280; doi:10.1371/journal.pone.0037974)

## Slide 1
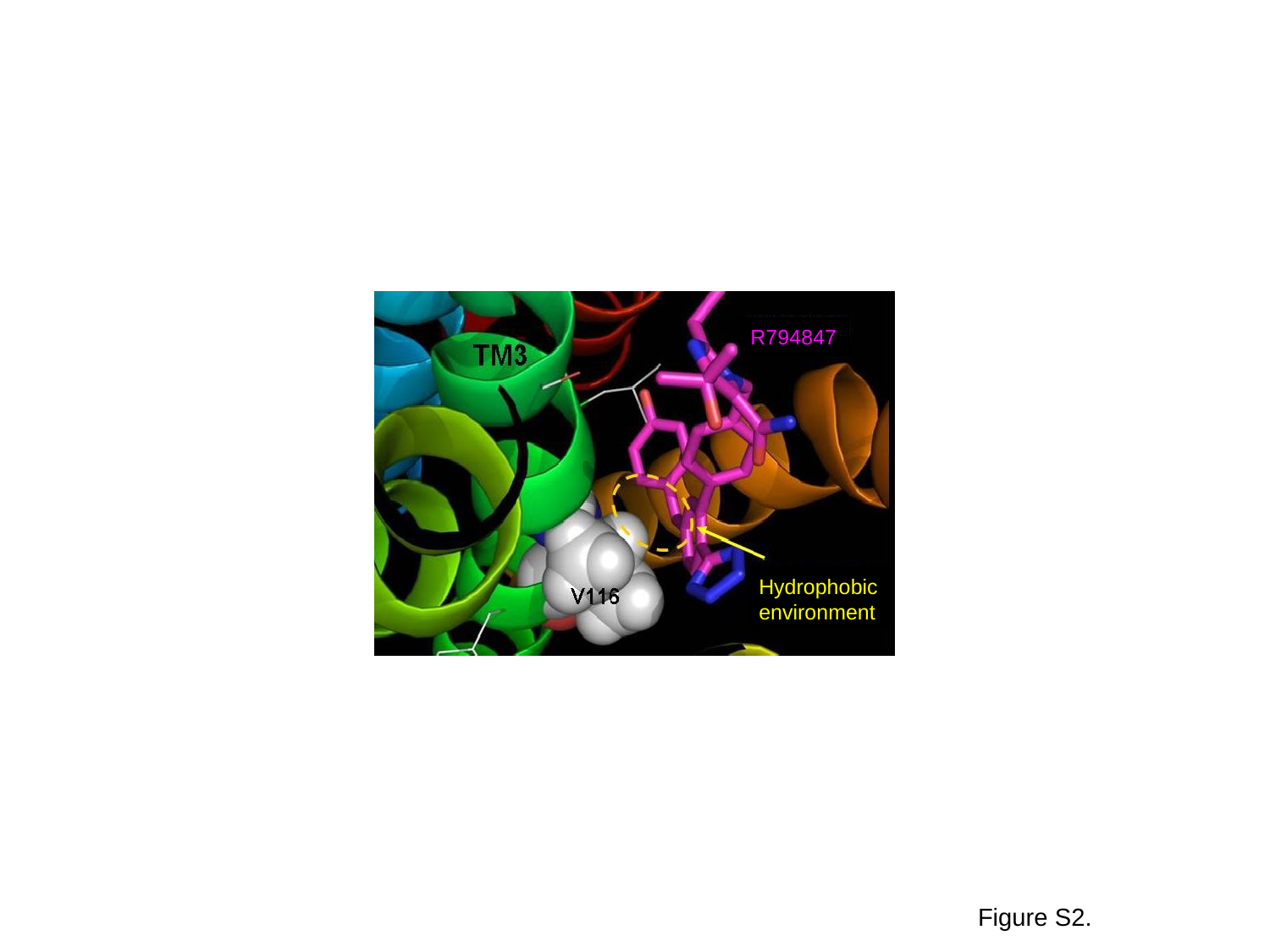

R794847
Hydrophobic
environment
Figure S2.

Supplement: Figure S2 — Putative binding mode of R794847 and Val116 of AT1 receptor. Transmenbrans (TMs) are shown as colored ribbons: green (TM3), lime green (TM4), yellow (TM5) and orange (TM6). Yellow dots indicate a hydrophobic environment. (PPT) [file pone.0037974.s002.ppt]
