# Supplementary material for: Small Molecules with Similar Structures Exhibit Agonist, Neutral Antagonist or Inverse Agonist Activity toward Angiotensin II Type 1 Receptor
Source: PLoS One. 2012 Jun 14;7(6):e37974. doi: 10.1371/journal.pone.0037974 (PMC3375280; doi:10.1371/journal.pone.0037974)
Supplement: Table S1 — Maximal binding capacities ( B max) of Ang II to AT1 wild-type (WT) and mutants receptors. (DOC) [file pone.0037974.s003.doc]

**Table S1. Maximal binding capacities (*B*_max_) of Ang II to AT_1_ wild-type (WT) and mutant receptors**

*B*_max_ *B*_max_

Receptor (pmol/mg protein) Receptor (pmol/mg protein)

WT 0.61±0.05 S105A 0.38±0.05

C76A/C289A 0.65±0.06 S106G 0.59±0.14

C76A/A106C/C289A 0.54±0.03 S107A 0.85±0.25

C76A/S107C/C289A 0.65±0.07 V108A 0.52±0.08

C76A/V108C/C289A 0.47±0.09 S109A 0.52±0.05

C76A/S109C/C289A 0.56±0.10 F110A 0.61±0.18

C76A/F110C/C289A 0.40±0.02 N111G 0.47±0.04

C76A/N111C/C289A 0.35±0.05 V112A 0.47±0.03

C76A/V112C/C289A 0.46±0.06 Y113F 0.59±0.09

C76A/Y113C/C289A 0.29±0.07 Y113A 0.29±0.02

C76A/A114C/C289A 0.62±0.11 A114G 0.42±0.15

C76A/S115C/C289A 0.43±0.09 S115A 0.32±0.16

C76A/V116C/C289A 0.54±0.09 V116A 0.74±0.05

C76A/F117C/C289A 0.54±0.06 F117A 0.45±0.13
